# Interrupted time series analysis using autoregressive integrated moving average (ARIMA) models: a guide for evaluating large-scale health interventions

**DOI:** 10.1186/s12874-021-01235-8

**Published:** 2021-03-22

**Authors:** Andrea L. Schaffer, Timothy A. Dobbins, Sallie-Anne Pearson

**Affiliations:** 1 Centre for Big Data Research in Health, UNSW Sydney, Level 2, AGSM Building, Sydney, Australia; 2 School of Public Health and Community Medicine, UNSW Sydney, Sydney, Australia; 3 Menzies Centre for Health Policy, University of Sydney, Sydney, Australia

**Keywords:** Interrupted time series analysis, Autoregressive integrated moving average models, Policy evaluation, Intervention analysis

## Abstract

**Background:**

Interrupted time series analysis is increasingly used to evaluate the impact of large-scale health interventions. While segmented regression is a common approach, it is not always adequate, especially in the presence of seasonality and autocorrelation. An Autoregressive Integrated Moving Average (ARIMA) model is an alternative method that can accommodate these issues.

**Methods:**

We describe the underlying theory behind ARIMA models and how they can be used to evaluate population-level interventions, such as the introduction of health policies. We discuss how to select the shape of the impact, the model selection process, transfer functions, checking model fit, and interpretation of findings. We also provide R and SAS code to replicate our results.

**Results:**

We illustrate ARIMA modelling using the example of a policy intervention to reduce inappropriate prescribing. In January 2014, the Australian government eliminated prescription refills for the 25 mg tablet strength of quetiapine, an antipsychotic, to deter its prescribing for non-approved indications. We examine the impact of this policy intervention on dispensing of quetiapine using dispensing claims data.

**Conclusions:**

ARIMA modelling is a useful tool to evaluate the impact of large-scale interventions when other approaches are not suitable, as it can account for underlying trends, autocorrelation and seasonality and allows for flexible modelling of different types of impacts.

**Supplementary Information:**

The online version contains supplementary material available at 10.1186/s12874-021-01235-8.

## Background

Before and after study designs are often used to quantify the impact of population-level health interventions on processes of care and population-level health outcomes. They rely on the “natural experiment” resulting from implementing interventions, dividing time into “pre-intervention” and “post-intervention” periods. However, observational studies relying on a small number of measurements pre- and post-intervention are prone to bias as they do not account for pre-existing underlying short- and long-term trends [1]. In contrast, interrupted time series (ITS) analysis (also called “intervention analysis”) is more robust as it does control for these issues by longitudinally tracking the outcome before and after an intervention. ITS is considered one of the best designs for establishing causality when randomised controlled trials (RCTs) are neither feasible nor ethical [2, 3]. In fact, when combined with a control series, ITS designs often generate similar results to RCTs [4].

Several published papers have addressed the topic of using ITS approaches to evaluate health interventions [5–9]. However, these have focussed primarily on segmented regression, the simplest form of ITS analysis. Segmented regression models use time as a predictor variable; a simple segmented regression model can be expressed as:

$$ {Y}_t=\alpha +{\beta}_1\times time+{\beta}_2\times intervention+{\beta}_3\times time\kern0.5em since\kern0.5em intervention+{\varepsilon}_t $$

Where *Y*_*t*_ is the outcome at a given time point (*t*), the *time* variable represents time since start of the study period, the *intervention* variable indicates whether the time *t* is before (0) or after (1) the implementation of the intervention, and the *time since intervention* variable represents time elapsed since intervention implementation, taking a value of 0 prior to the intervention. A key assumption of linear regression is that the errors (residuals) are independent and not correlated. However, this assumption is often violated with time series.

The segmented regression approach is most appropriate when a time series has a linear or otherwise easily modelled trend and independently distributed residuals. In practice, patterns in data can be unclear or difficult to identify, with considerable variation. Thus, some time series may not be amenable to segmented regression due to the difficulty in modelling the autocorrelation structure. One alternative to segmented regression is Autoregressive Integrated Moving Average (ARIMA) models. ARIMA models differ from segmented regression in that the outcome *Y*_*t*_ is regressed only on the outcome measured at previous time points (not on time itself). However, there is little guidance in the literature about how to fit these models in the context of ITS analysis. Given the quantity and complexity of health data now being collected and made available for research, ARIMA has become an increasingly useful tool for researchers interested in evaluating large-scale interventions.

In this paper we will describe the underlying theory behind ARIMA models and how they can be used to evaluate population-level interventions, such as the introduction of health policies, illustrated using an example of the introduction of a health policy to deter inappropriate prescribing of quetiapine, an antipsychotic, in Australia.

## Methods

### Time series properties

A time series is a sequence of data points at equally spaced points in time and ordered chronologically. Time series typically exhibit three features: non-stationarity, autocorrelation, and seasonality.

#### Non-stationarity

A requirement of ARIMA modelling is that the time series is *stationary.* A stationary series has three properties: a constant mean; constant variance; and constant covariance that depends only on the time interval between values. A stationary series (also called a “white noise process”) is easier to analyse as it can be modelled with fewer parameters. While it may fluctuate, it will always revert to a constant mean and is thus easier to predict. There are two main sources of non-stationarity: the first is changing variance over time (heteroscedasticity) which can often be addressed by applying a log transformation; and the second is an increasing or decreasing trend which can often be eliminated by taking the first difference (i.e. *Y*_*t*_ − *Y*_*t* − 1_). Occasionally a second differencing may be required to achieve stationarity, but third-order differencing and above is rare [10]. To be exact, the above definition is for a *weakly* stationary series. A time series is considered *strictly stationary* if the probability distribution of a sequence of observations is unchanged by shifts in time. Strictly stationary series are rare, and it is often enough to assume *weak stationarity*.

#### Autocorrelation

Time series observations are often correlated with observations at previous time points and are thus not independently distributed. This correlation is referred to as autocorrelation or serial correlation. As previously mentioned, time series exhibiting autocorrelation do not satisfy standard regression analysis assumptions. As autocorrelated data are typically not stationary, differencing the data is often enough to remove autocorrelation and therefore any necessary data transformations should be performed before testing for autocorrelation.

Autocorrelation functions (ACFs) can be used to check for stationarity and autocorrelation. An ACF plots the correlation between each observation and previous values at various lags, where a lag is the number of time points between an observation and its previous values. The companion to the ACF is the partial ACF (PACF), which is the correlation between an observation and past values that is not explained by correlations at lower order lags. For instance, the PACF value at lag 4 is the correlation between an observation (*Y*_*t*_) and the previous observation at lag 4 (*Y*_*t* − 4_), after adjusting for the correlation between *Y*_*t*_ and *Y*_*t* − 3_, *Y*_*t* − 2_, and *Y*_*t* − 1_. For a stationary series, the autocorrelation in the ACF plot should decay quickly; with a non-stationary series, the ACF will decay slowly.

#### Seasonality

Seasonality refers to variation of a fixed or known frequency, occurring at regular time intervals, such as time of year or day of the week. Seasonality in time series of health data is common and can be due to natural causes, such as weather patterns, or business/administrative processes such as weekend or holiday effects. For instance, antibiotic prescriptions and influenza hospitalisations are more common in the winter months [11, 12]. Further, in some jurisdictions medicine dispensings are highest at the end of a calendar or financial year due to the financial incentives to stockpile medicines [13, 14]. The extent of seasonality will depend on the unit of time of the series; for instance, seasonality is rare in time series measured at yearly intervals.

With seasonal monthly data, there will likely be significant autocorrelation at lag 12 in the ACF plot. In ARIMA modelling, seasonality is usually dealt with by taking the *seasonal difference*. That is, with monthly data, you take the difference between each observation and the previous value at lag 12 (*Y*_*t*_ − *Y*_*t* − 12_). For quarterly data, you would use lag 4. Note that when taking the seasonal difference for monthly data, the first 12 observations are lost, since the seasonal difference cannot be calculated for those observations. This is important to keep in mind – if you have seasonal data, in general you will need more time points in your series to adequately control for seasonal effects.

### Components of ARIMA models

ARIMA models have a single dependent variable (*Y*_*t*_) that is a function of past values of *Y* and the error term (*ϵ*_*t*_). As ARIMA models assume that errors are normally distributed, they can accommodate any continuous outcome (such as rates or means), as well large counts that are not bounded by zero. While ARIMA cannot be used with small counts that follow a Poisson distribution, in recent years approaches to modelling serially correlated count data have been developed using generalised linear models [15, 16]. Before getting into full ARIMA models, we introduce the basic components.

1. *Autoregressive (AR) model*: *Y*_*t*_ is predicted by one or multiple lagged values of *Y*_*t*_. This is represented by the equation below, where *c* is a constant, *ϕ* is the magnitude of the autocorrelation, *p* is the number of lags, and *ϵ*_*t*_ is the error.
  $$ {Y}_t=c+{\phi}_1{Y}_{t-1}+{\phi}_2{Y}_{t-2}+\dots +{\phi}_p{Y}_{t-p}+{\epsilon}_t $$
2. *Moving average (MA) model*: *Y*_*t*_ is predicted by one or multiple lagged values of the error (*ϵ*_*t*_). This is not to be confused with moving average smoothing. In the equation below, *θ* is the value of the autocorrelation of the errors, and *q* is the number of lags.
  $$ {Y}_t=c+{\theta}_1{\epsilon}_{t-1}+{\theta}_2{\epsilon}_{t-2}+\dots +{\theta}_q{\epsilon}_{t-q} $$
3. *Seasonal model*: *Y*_*t*_ is predicted by lagged values of *Y*_*t*_ at a regular interval *s* (the season). In the equation below, *Ф* is the value of the autocorrelation, and *s* is the seasonality (e.g. 52 for weekly, 12 for monthly, 4 for quarterly). Seasonal models will also often require differencing, as well as autoregressive and/or moving average terms.
  $$ {Y}_t=c+\varPhi {Y}_{t-s}+{\epsilon}_t $$
4. *Differencing (Integration):* In an ARIMA model, the time series being modelled must be stationary to obtain meaningful predictions. Stationarity is induced by differencing, which refers to calculating the difference between adjacent observations.
  $$ {Y}_t^{\prime }={Y}_t-{Y}_{t-1} $$

An ARIMA model is a combination of an AR model, MA model, and differencing (Integration). If *ϕ* = 0 and *θ* = 0 and *Ф* = 0 then the time series is a white noise process expressed as *Y*_*t*_ = *c* + *ϵ*_*t*_ where *c* is a constant.

The basic notation for describing a non-seasonal ARIMA model is (*p*, *d*, *q*), where *p*, *d*, and *q* are positive integers:

- p = the order of the AR part of the model;
- d = the degree of non-seasonal differencing; and
- q = the order of the MA part of the model.

For example, a white noise (stationary) model is ARIMA (0, 0, 0). An AR model with *p* lags is ARIMA(*p*, 0, 0), and an MA model with *q* lags is ARIMA (0, 0, *q*). If there is seasonality, the ARIMA model is expressed as: (*p*, *d*, *q*) × (*P*, *D*, *Q*)_*S*_. Here, *D* is the degree of seasonal differencing, and *P* and *Q* are the AR and MA terms for the seasonal component.

### Evaluating interventions using ARIMA

The aim of ITS analysis when used to evaluate interventions is to estimate the impact of the intervention’s implementation on a given outcome, or in other words the “intervention effect”. While there is a wide variety of impacts that may be observed, here we will focus on three main types: step change, pulse and ramp. If we use *T*_0_ to represent the starting time of the intervention, these are summarised as:

- *Step change (also called a level shift)*: A sudden, sustained change where the time series is shifted either up or down by a given value immediately following the intervention. The step change variable takes the value of 0 prior to the start of the intervention, and 1 afterwards.
  $$ {S}_t=\left\{\begin{array}{c}0, if\ t<{T}_0\\ {}1, if\ t\ge {T}_0\end{array}\right. $$
- *Pulse*: A sudden, temporary change that is observed for one or more time points immediately after the intervention and then returns to baseline level. The pulse variable takes the value of 1 on the date of the intervention, and 0 otherwise.
  $$ {P}_t=\left\{\begin{array}{c}0, if\ t\ne {T}_0\\ {}1, if\ t={T}_0\end{array}\right. $$
- *Ramp*: A change in slope that occurs immediately after the intervention. The ramp variable takes the value of 0 prior to the start of the intervention and increases by 1 after the date of the intervention.
  $$ {R}_t=\left\{\begin{array}{c}0,\kern.3em if\kern0.5em t<{T}_0\\ {}t-{T}_0+1,\kern.3em if\kern0.5em t\ge {T}_0\end{array}\right. $$

Ideally, the potential shape of the intervention impact should be hypothesised a priori. The shape depends on several factors, including the nature of the intervention, such as whether it is temporary or ongoing, and the specific outcome being assessed. For instance, in our 2015 study [17] we evaluated the impact of negative media around use of statin medicines and found that this temporary event resulted in both a temporary increase in statin discontinuation (a “pulse”) but a sustained decrease in statin dispensing (a “step change”). Ongoing or permanent interventions, such as increased restrictions on prescribing of a medicine [18] or introduction of plain packaging on tobacco products [19] are more likely to have long-term effects, although these may be immediate or gradual (a “ramp”). For some interventions, the change is best represented by a combination of impact variables; for instance, it is common for there to be both a step change and change in slope (ramp). If there are multiple potential models, the Akaike information criterion (AIC) and/or Bayesian information criterion (BIC) can be used to select the most appropriate combination of impact variables.

It is also important to consider whether changes may occur prior to the implementation of the intervention; for example, when it was announced that there would be increased restrictions placed on prescribing of alprazolam in Australia, prescribing of this medicine started declining in anticipation of this change [18]. Lastly, in some cases, the impact may be suspected to be delayed by one or more time units. We recommend prespecifying a reasonable period of time in which it would be expected for the impact to be observed based on content knowledge or previous research to avoid spurious associations. The most appropriate delay within this range of options can be determined at the modelling stage [20].

In ITS analysis, ARIMA forecasts *Y*_*t*_ in the absence of the intervention (the “counterfactual”) and determines how the observed diverges from this forecast. Unlike segmented regression, including time or seasonal dummy variables in the ARIMA model is not necessary, as ARIMA can eliminate trends and seasonality through differencing. If the trend is eliminated via differencing then the pre- and post-intervention trends cannot be estimated from the model. However, if estimation of the pre- and/or post-intervention slope is desired, this can be accommodated by including time as a covariate and incorporating AR and MA terms to address autocorrelation (e.g. ARMA models) [21, 22].

### Fitting an ARIMA model

**Fig. 1:**
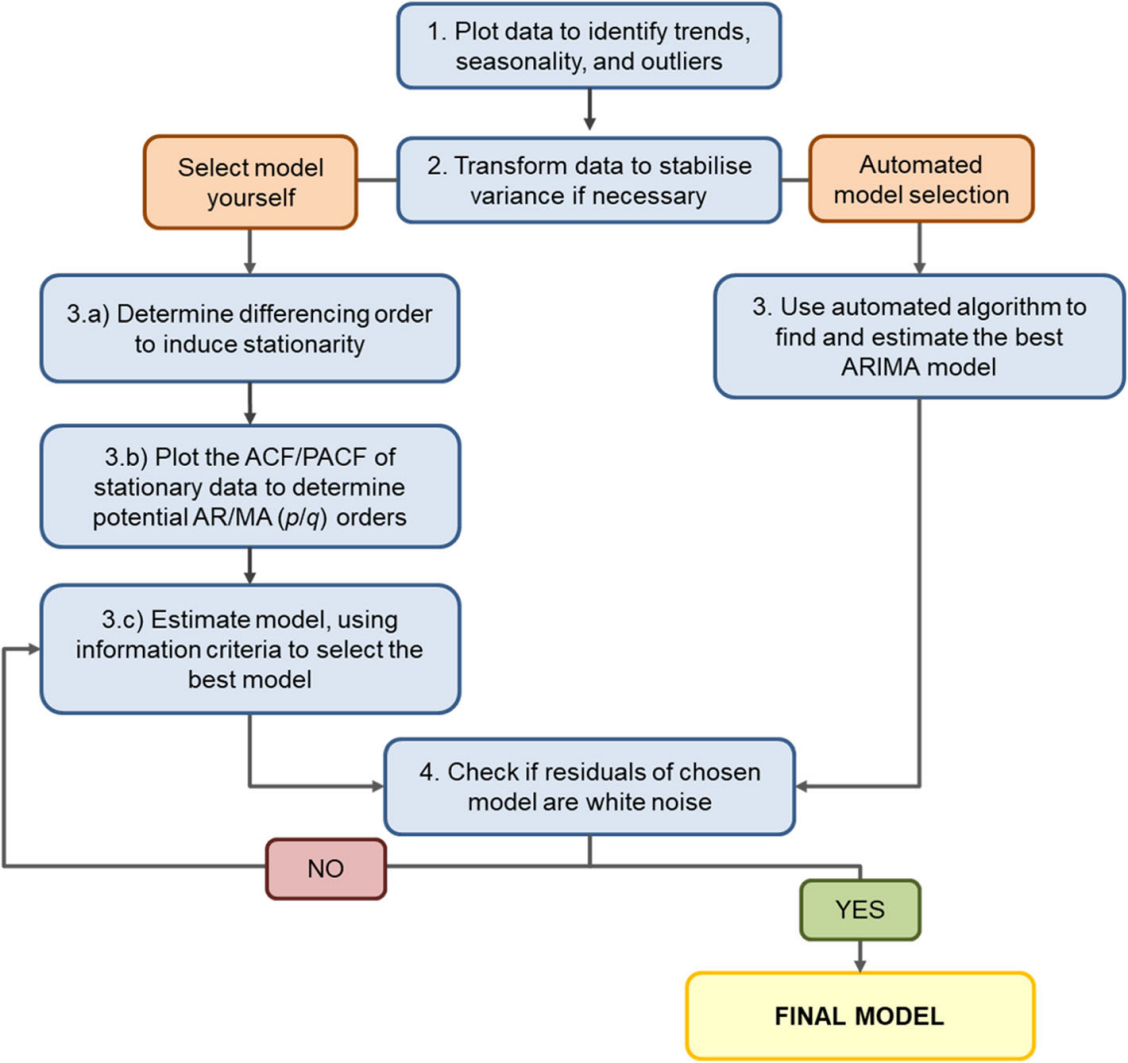
Flow chart for ARIMA model selection. Adapted from Hyndman and Athanasopoulos [10].

The next step is determining the parameters of the ARIMA model. A common approach is called the Box-Jenkins method, involving model identification and selection, parameter estimation, and model checking [23]. There now exist automated algorithms in statistical packages (such as R) that simplify the process by identifying the best fitting ARIMA model based on minimising the information criteria (AIC, BIC). However, we also describe the manual process below, illustrated in Fig. 1.

1. **Plot data to understand patterns**: Before proceeding to model fitting, plot the time series to understand the patterns, specifically pre-existing trends, seasonal effects, and extreme or outlier values. If outliers are present, how to deal with will depend on their cause and influence on the model and the recommendations are the same for ARIMA as for other regression models. For instance, if the researchers are aware that these extreme values are due to external factors, such as other interventions or known misclassification, these should be explicitly modelled in the data.
2. **Transform data to stabilise variance (if necessary).** If the variance is changing over time, a log-transformation should be applied.
3. **Model selection**: While automated algorithms in several statistical packages can identify candidate *p* and *q* parameters, they can sometimes be estimated based on the ACF/PACF plots.
  - **Determine differencing order to induce stationarity**: If there is a trend, a first order difference is required and *d* = 1. If there is seasonality, a seasonal difference is required and *D* = 1. The ACF plot or unit-root tests (e.g. Dickey-Fuller test) can also be used to help identify whether the time series is stationary and whether differencing will be required. Most automated algorithms allow you to prespecify the *d* and *D* terms in the model.
  - **Plot the ACF/PACF of stationarity data to determine potential AR/MA orders**: After the time series has been made stationary by transformation and/or differencing, next determine which AR (*p*/*P*) or MA (*q*/*Q*) orders are needed to correct for remaining autocorrelation. If the stationary series has positive autocorrelation at lag 1, then AR terms typically are needed. If the autocorrelation is negative at lag 1, then the model may need MA terms. Usually models will require only AR terms *or* MA terms, rarely both. However, it is not always straightforward. Table 1 includes guidance on selecting the most appropriate AR and MA terms.
  - **Estimate model and use information criteria to find the best model**: Estimate your model, using the *p*, *d*, *q*, *P*, *D*, and *Q* terms identified previously, and use information criteria (AIC, BIC) to help identify the best model. If an automated algorithm is used to select the terms, it should be viewed as a tool only, as it does not guarantee a well-fitting model.
4. **Check if residuals of chosen model are white noise.** This can be done by looking at residual plots and by formally testing for the presence of autocorrelation by using the Ljung-Box test for white noise. If autocorrelation is still present in the residuals or your model is otherwise a poor fit, then choose different AR and/or MA orders. If the data have not previously been transformed, a transformation may help with non-normally distributed residuals. In general, determining the AR and MA terms is an iterative process, involving trial and error. Importantly, there may not be one “right” model. The aim is to select the most parsimonious model (i.e. smallest *p*/*P* and *q*/*Q*) that has a good fit and adequately controls for autocorrelation and seasonality. Once the final ARIMA model is selected, the intervention impact can be estimated.

**Table 1:** Tips for selecting most appropriate autoregressive (p) and moving average (q) terms from autocorrelation and partial autocorrelation

Model type	Characteristics of ACF and PACF	
	ACF	PACF
ARIMA(p,d,0)	Tails off or is sinusoidal	Cuts off lag *p*
ARIMA(0,d,q)	Cuts off lag *q*	Tails off or is sinusoidal
ARMA(p,d,q)	Tails off or is sinusoidal	Tails off or is sinusoidal

### Transfer functions

Another advantage of ARIMA models is the ability to move beyond the basic intervention impact shapes and model more complex impacts via “transfer functions”. Transfer functions describe the relationship between the intervention and the outcome series *Y*_*t*_. They modify the relationship between the above inputs (step change, pulse, ramp) and the time series to model more complex relationships, such as gradual level shifts, or a pulse that decays gradually over time, and can also incorporate lagged effects. The general form of a transfer function is $\frac{\omega (B)}{\delta (B)}$, or:

$$ {Y}_t=\mu +\frac{\omega_0+{\omega}_1B+{\omega}_2{B}^2+\cdots +{\omega}_h{B}^h}{1-{\delta}_1B-{\delta}_2{B}^2-\cdots -{\delta}_r{B}^r}{X}_t+{\varepsilon}_t $$

where *B* is the backshift operator (i.e. *B*^*p*^*Y*_*t*_ = *Y*_*t* − *p*_). In the transfer function, *ω*_0_ represents the initial value for the impact of the intervention at the time of the intervention (*T*), *δ* is the decay rate, *X*_*t*_ is the intervention variable (step change, pulse, or ramp). The values of *h* and *r* must be specified by the researcher; *h* describes when the effect happens, while *r* represents the decay pattern. Model fit statistics (such as AIC and BIC) can help determine the most appropriate form for the transfer function as well as the timing of the event (i.e. if the impact was delayed and if so by how much). Table 2 describes the most common scenarios, using the intervention indicator variables described above, and where *h* = 0, and *r* = 0 or *r* = 1. The use of transfer functions is a complex topic, and several texts cover them in more detail [23–25].

**Table 2:** Description of transfer functions for interrupted time series analysis in ARIMA

Function	Values for ***h*** and ***r***	Transfer function	Response ***i*** at times 0 through ***k*** post-intervention	Form of response	Interpretation
**Step function** $$ {S}_t=\left\{\begin{array}{c}0, if\ t<T\\ {}1, if\ t\ge T\end{array}\right. $$	*h* = 0, *r* = 0	*ω*_0_	*i*_0_ = *ω*_0_ *i*_1_ = *ω*_0_ *i*_2_ = *ω*_0_ … *i*_*k*_ = *ω*_0_		The time series increases by *ω*_0_ immediately following the intervention, and remains at this new level for the duration of the study period.
	*h* = 0, *r* = 1	$\frac{\omega_0}{\left(1-{\delta}_1B\right)}$ (|*δ*_1_| < 1)	*i*_0_ = *ω*_0_ *i*_1_ = *ω*_0_(1 + *δ*_1_) ${i}_2={\omega}_0\left(1+{\delta}_1+{\delta}_1^2\right)$ … ${i}_k={\omega}_0\left(1+{\delta}_1+{\delta}_1^2+\dots +{\delta}_1^k\right)$		The time series increases by *ω*_0_ immediately following the intervention, and increases by ${\omega}_0{\delta}_1^k$ each subsequent time point until it reaches a new level, calculated by $\frac{\omega_0}{\left(1-{\delta}_1\right)}$.
**Pulse function** $$ {P}_t=\left\{\begin{array}{c}0, if\ t\ne T\\ {}1, if\ t=T\end{array}\right. $$	*h* = 0, *r* = 0	*ω*_0_	*i*_0_ = *ω*_0_ *i*_1_ = 0 *i*_2_ = 0 … *i*_*k*_ = 0		The time series increases by *ω*_0_ immediately following the intervention and returns to baseline immediately afterwards.
	*h* = 0, *r* = 1	$\frac{\omega_0}{\left(1-{\delta}_1B\right)}$ (|*δ*_1_| < 1)	*i*_0_ = *ω*_0_ *i*_1_ = *ω*_0_*δ*_1_ ${i}_2={\omega}_0{\delta}_1^2$ … ${i}_k={\omega}_0{\delta}_1^k$		The time series increases by *ω*_0_ the time of the intervention, and decays by (1 − *δ*_1_) each subsequent time point.
**Ramp function** $$ {R}_t=\left\{\begin{array}{c}0, if\ t<T\\ {}t-T+1, if\ t\ge T\end{array}\right. $$	*h* = 0, *r* = 0	*ω*_0_	*i*_0_ = *ω*_0_ *i*_1_ = 2*ω*_0_ *i*_2_ = 3*ω*_0_ … *i*_*k*_ = (*k* + 1)*ω*_0_		The time series increases by *ω*_0_ at each time point.

### Incorporation of a control series

Including a control series in ITS analysis improves causal inference, as ITS cannot exclude the possibility that any observed change was due to the intervention of interest, or another co-intervention or event. A control series is one that is not impacted by the intervention; selection of an appropriate control is described elsewhere [3]. As with ITS in segmented regression, including a control series involves running an ARIMA model for the series of interest, and separately for the control series [17]. If a change is observed in the intervention series but not the control series, this provides evidence that the impact was specific to the intervention.

### Sample size requirements

There is no definitive guidance on how many time points are required to apply ARIMA modelling. The oft-quoted value of a minimum of 50 time points is based on a statement by Box and Jenkins, [23] but this has no empirical basis and has not been tested formally. In reality, a one-size-fits-all approach is simplistic. The more variable and noisier the data, the more observations will be needed to distinguish the underlying patterns from the noise. In uncomplicated cases, ARIMA can perform satisfactorily with short time series, as long as there are enough time points to estimate all parameters [26]. In the presence of seasonality, there should be enough time points to identify the seasonal effects and to account for seasonal differencing.

## Results

### Data and context

Here we demonstrate the use of ARIMA modelling to quantify the impact of a health policy intervention, using Australian medicine dispensing claims. The policy restricted the conditions under which quetiapine, an antipsychotic medicine, could be subsidised (data, R code, and SAS code are included in **Additional Files** **1****,** **2** and **3** respectively).

Prior to January 1, 2014, new prescriptions for the lowest quetiapine tablet strength (25 mg) could include up to 5 refills, meaning patients could have their prescription refilled up to 5 times before returning to their doctor for a new prescription. However, due to growing concerns about inappropriate prescribing, after January 1, 2014 new prescriptions for this tablet strength could not include refills [27]. Our primary outcome was the number of monthly dispensings of 25 mg quetiapine, of which we had 48 months of observations (January 2011 to December 2014).

In Australia, medicine dispensing claims have significant yearly seasonality [13]. Medicines are subsidised for citizens and eligible residents through the Pharmaceutical Benefits Scheme (PBS), with people paying an out-of-pocket co-payment towards the cost of their medicines, while the remainder is subsidised. If a person’s (or family’s) total out-of-pocket costs reach the “Safety Net threshold” for the calendar year, they are eligible for a reduced co-payment for the remainder of that year. Thus, there is an incentive for people reaching their Safety Net to refill their medicines more frequently towards the end of the year. Hence, we see an increase in prescriptions at the end of the year, followed by a decrease in January.

For the change in dispensing of 25 mg quetiapine, due to the nature of the intervention we postulated there would be an immediate drop in dispensings post-intervention (step change), as well as a change in slope (ramp). Thus, we included variables representing both types of impacts in our model. For both impacts, *h* = 0 and *r* = 0.

### Steps 1 and 2: plot data and transform if necessary

**Fig. 2:**
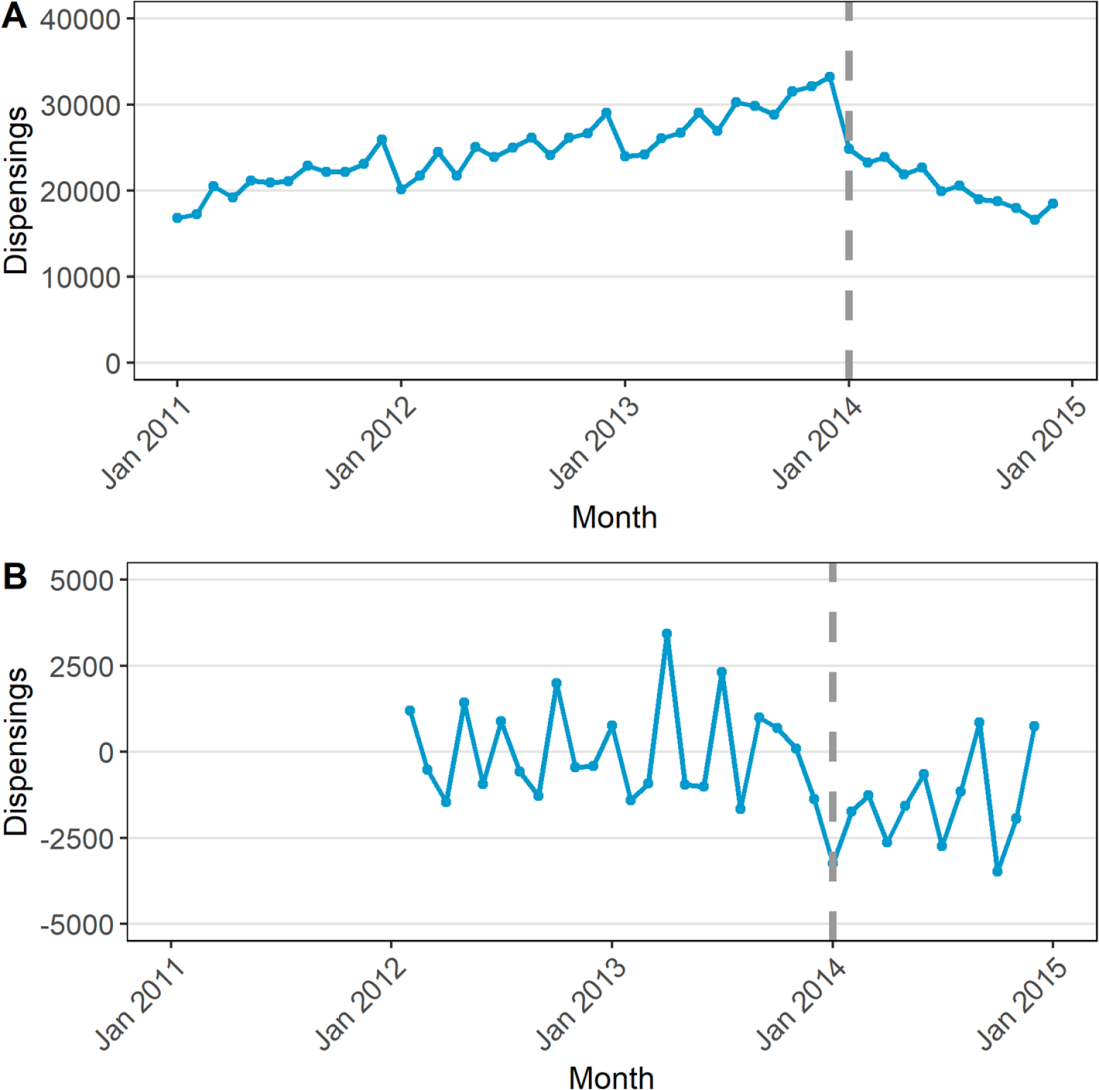
Monthly dispensings of the 25 mg strength quetiapine (A) and the series after first order and seasonal differencing (B)

The data are plotted in Fig. 2a, where we observe that due to the Safety Net effect discussed above, dispensings are higher in December, and lower in January [13]. As the variance appears stable over time, no data transformation is needed.

### Step 3: select model

**Fig. 3:**
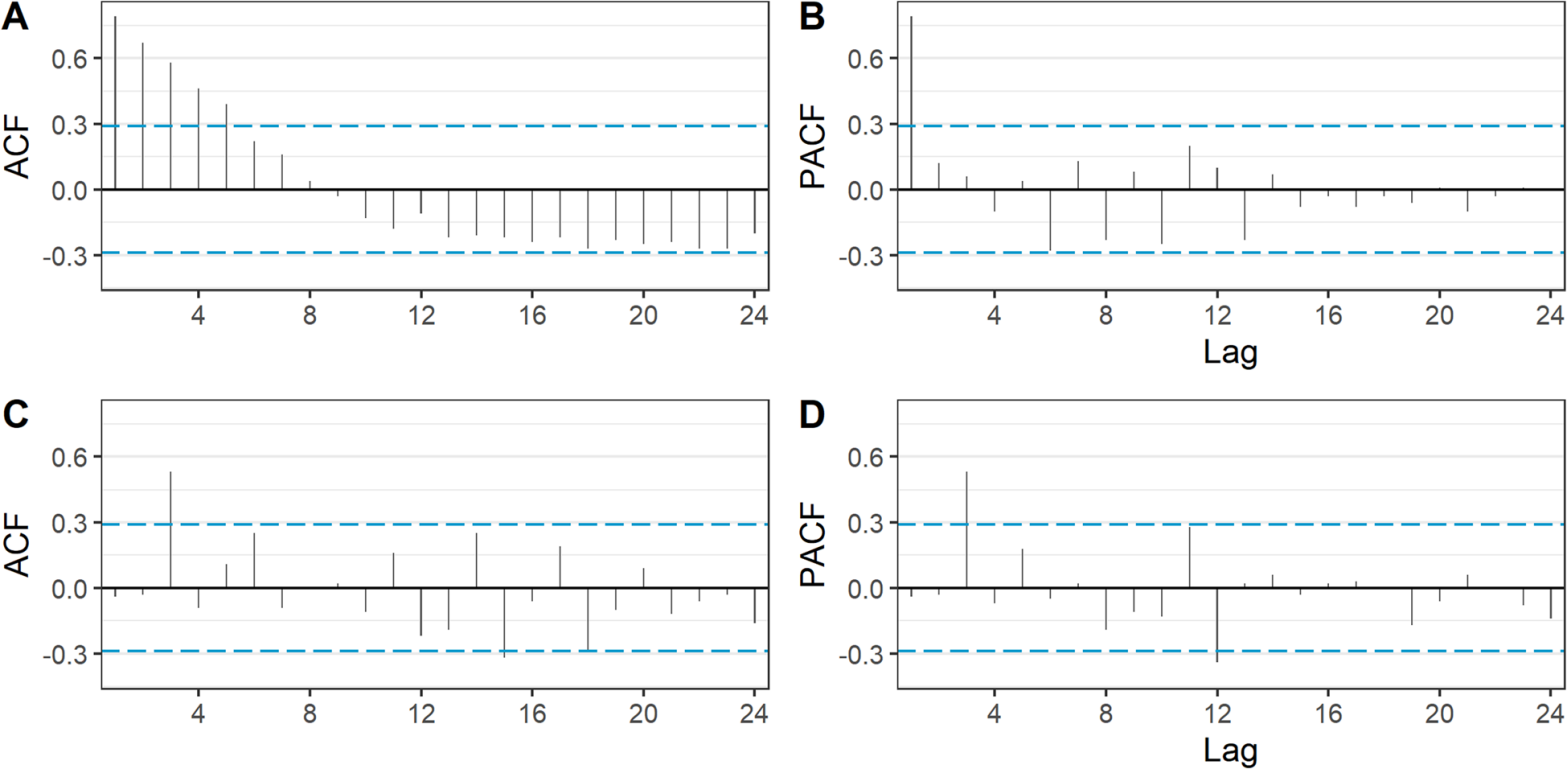
Autocorrelation and partial autocorrelation function (ACF and PACF) plots, prior to differencing (A and B) and after differencing (C and D)

To help induce stationarity, we determined that a first difference (*d*) was needed due to the visible increasing trend prior to the subsidy change, and that a seasonal difference (*D*) was needed due to the seasonality of the series. Figure 2b shows the series after these differences have been applied, with the trend eliminated. As the seasonal difference cannot be calculated for the first 12 observations as at least 13 observations are required to calculate the difference between *Y*_*t*_ and *Y*_*t* − 12_, the first year of data is not represented in the figure. The ACF and PACF plots are in **Fig.** **3**. In this figure, bars that fall above or below the dashed line represent statistically significant (*p* < 0.05) autocorrelation. In the ACF plot of the raw data, (Fig. 3a) we see significant autocorrelation that gradually dies off at lag 6. However, according to the PACF plot (Fig. 3b) the autocorrelation at higher lags is completely explained by autocorrelation at lower lags. We can also see that in Fig. 3c most of the autocorrelation has been removed just by differencing when compared with Fig. 3a.

In this case the ACF and PACF plots of the stationary (i.e. differenced) series are not particularly helpful in identifying the *p* and *q* parameters, as they do not fit any of the options in Table 1. Therefore, we used an automated algorithm, specifically *auto.arima()* in the *forecast* package for R, to identify the ARIMA model terms [28]. This algorithm iteratively searches over a series of potential ARIMA models for the one with the lowest AIC or BIC, with several constraints applied to avoid convergence problems. These include setting the maximum value of *p* and *q* to 5 and *P* and *Q* to 2, although these settings can be modified by the researcher if necessary. For our model, we pre-specified a value of *d* = 1 (to induce stationarity) and *D* = 1 (due to the presence of seasonality) but allowed the algorithm to select the most appropriate values for *p*, *d*, *P*, and *Q*.

The model with the lowest information criteria selected by the algorithm was (2,1,0) x (0,1,1)_12_. In other words, the autocorrelation order of the model (*p*) was 2, the moving average order of the model (*q*) was 0, the autocorrelation order of the seasonal part of the model (*P*) was 0, and the moving average order of the seasonal part of the model (*Q*) was 1. The model incorporates a first-order difference (*d* = 1) and a first-order seasonal difference (*D* = 1) to eliminate trend and induce stationarity. Thus, we will consider this as our potential final model.

### Step 4: check residuals

**Fig. 4:**
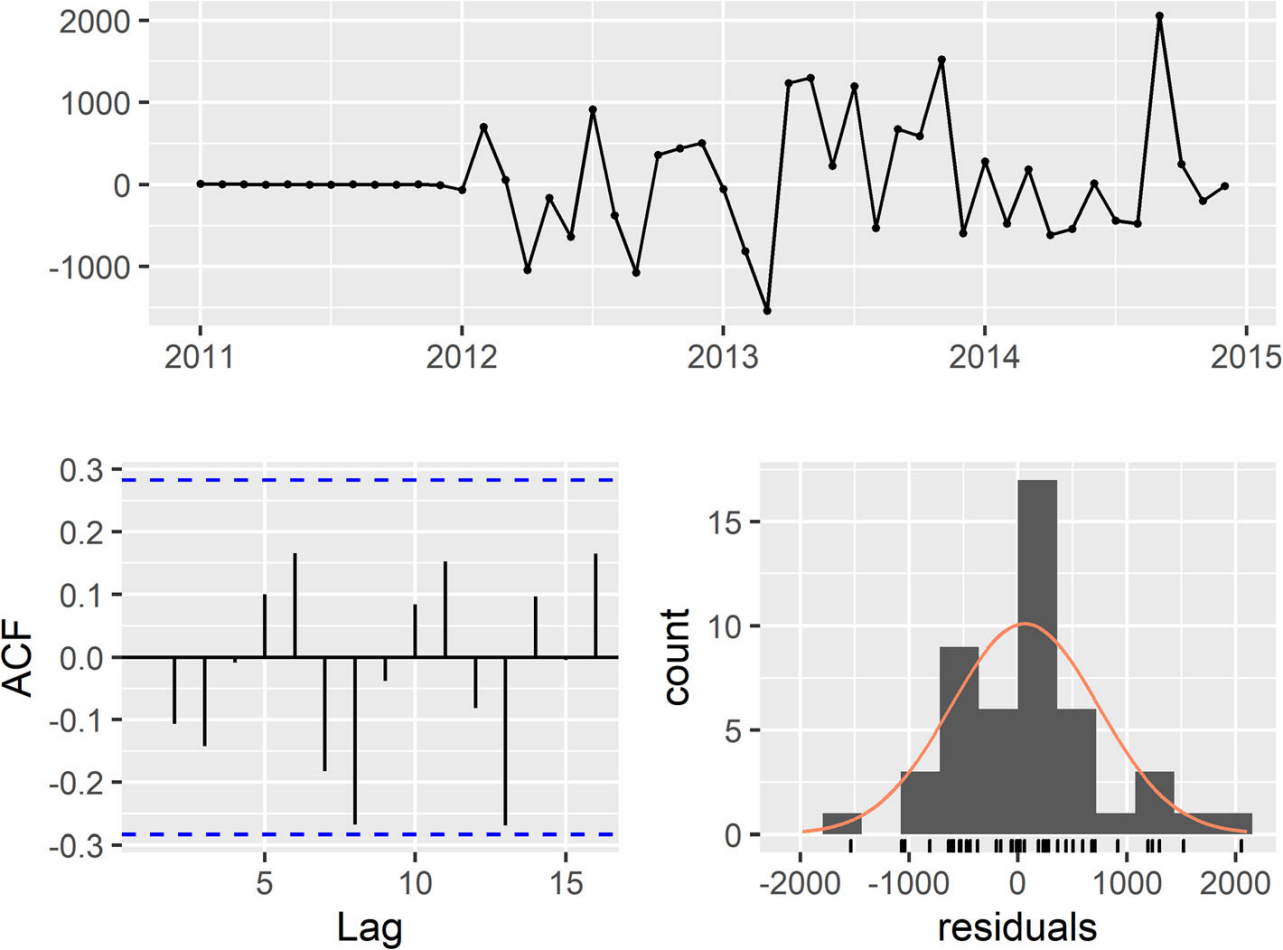
Residual check for final model, ARIMA (2,1,0)(0,1,1)_12_

The residual plots are in Fig. 4. There is no obvious pattern or significant autocorrelation in the residuals, and they are normally distributed. The *p*-value for the Ljung-Box test for white noise is 0.50 at 24 lags. As the null hypothesis for the Ljung-Box test is that there is no significant autocorrelation, we do not reject the null and our chosen model has a good fit.

### Final model

**Fig. 5:**
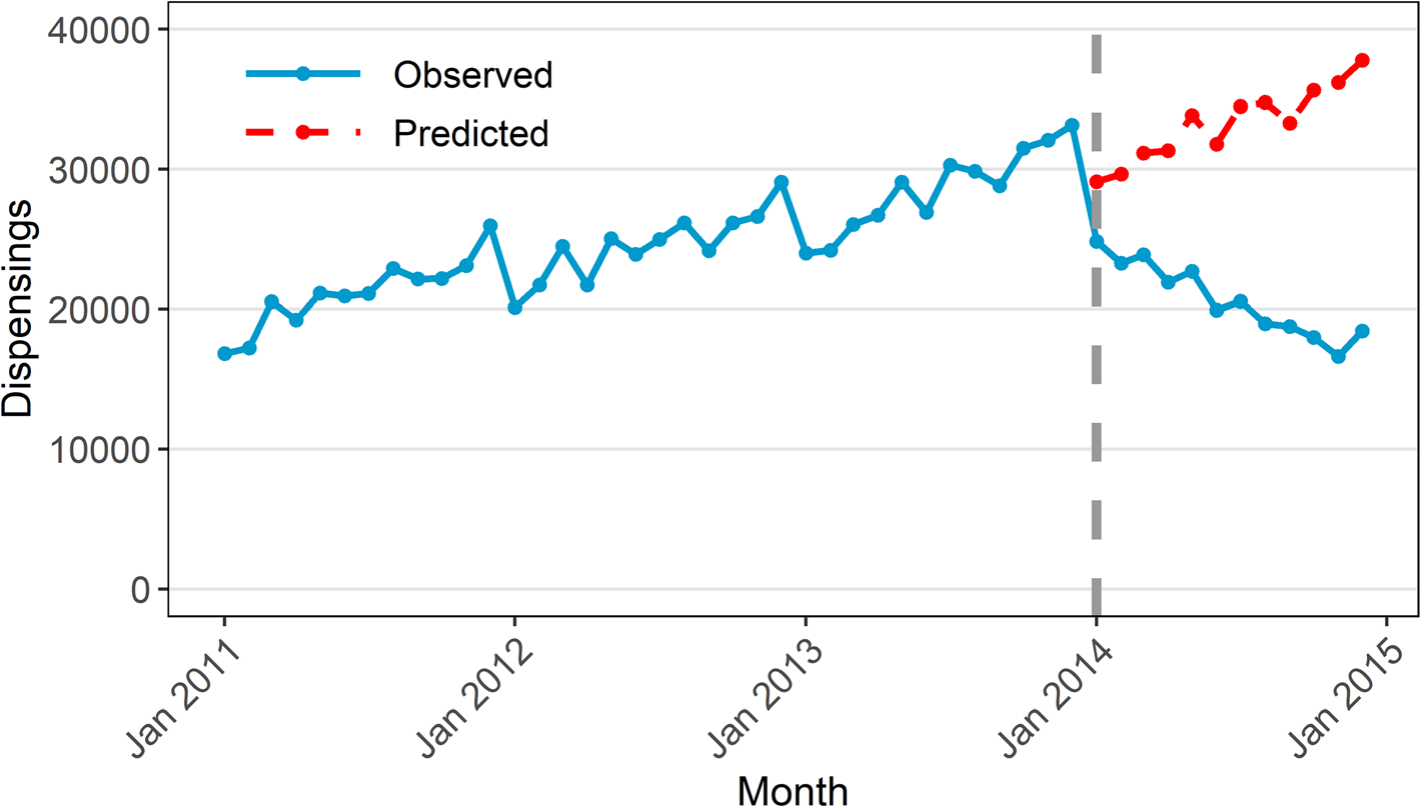
Observed values and predicted values in absence of intervention based on ARIMA model

The estimated step change was − 3285 dispensings (95% CI − 4465 to − 2104) while the estimated change in slope was − 1397 dispensings per month (95% CI − 1606 to − 1188). Figure 5 shows the values predicted by our ARIMA model in absence of the intervention (counterfactual) compared with the observed values. This means that the change in subsidy for 25 mg quetiapine in January 2014 was associated with an immediate, sustained decrease of 3285 dispensings, with a further decrease of 1397 dispensings every month. In other words, there were 4682 (3285 + 1397) fewer dispensings in January 2014 than predicted had the subsidy changes not been implemented. In February 2014, there were 6079 fewer dispensings (3285 + 2*1397). Importantly, our findings should only be considered valid for the duration of the study period (i.e. until December 2014).

## Discussion

Many health policies are implemented with a limited evidence base supporting their rationale, and even if well-intended can lead to unintended consequences [29, 30]. Thus, evaluation of health interventions is crucial to identify both intended and unintended impacts, to ultimately provide feedback to policy-makers and regulators, improve health care delivery, and inform future public health policy. However, many studies evaluating large-scale interventions use methods that are inadequate or poorly reported [31, 32]. As with all analyses, researchers interested in evaluating interventions should use fit-for-purpose tools for a particular research question, as relying on overly simplistic approaches can lead to misleading or biased results [1].

We have highlighted the importance of controlling for trends, seasonality, and autocorrelation. To a limited extent, segmented regression can also address these issues, typically by inclusion of time and season in the model as covariates, and often this will be enough to eliminate simple autocorrelation. In such cases, segmented regression may be preferred due to its ease of interpretability and implementation. However, there are circumstances in which segmented regression is inadequate. For instance, if the trend in the data is non-linear and/or had an irregular pattern, or if the seasonality is complex, such as weekly or daily, this can be difficult to capture in a segmented regression model. Lastly, if there is residual autocorrelation after running a segmented regression model then alternate approaches will need be considered, of which ARIMA is one.

At times, selecting the most appropriate ARIMA model can be challenging, time-consuming, and subjective, as traditional approaches that rely on ACF/PACF plots to identify model orders are often not informative, as seen in our example. However, there have been attempts over the years to automate the model selection process and simplify the process. We have applied one such algorithm, *auto.arima()* in the *forecast* package for R, which we have chosen due to its convenience and ease of use [28]. Such innovations have made ARIMA modelling more accessible, but as with all automated statistical approaches, still require a knowledgeable user to correctly apply and interpret the results.

It is important for researchers and analysts to have knowledge of a range of statistical tools that can be used as appropriate depending on the nature of the research question and data. ARIMA is one such tool; we have shown how ARIMA modelling can be used to evaluate health interventions when simpler approaches are not appropriate. While we have covered the foundations of ITS analysis using ARIMA models and the most common types of intervention impacts, there are other topics we have not touched on, such as use of cross correlation functions to identify delayed effects, the incorporation of covariates, and more complex transfer functions. These more complex topics have been covered in detail in other texts [23–25].

Despite the increasing use of ITS analysis, reporting of methods is highly variable and often inadequate [32, 33]. In a 2015 review, one third of studies did not report testing for autocorrelation and two thirds did not report adjusting for seasonality [33]. To maximise reproducibility, we encourage all researchers to publish code to ensure analyses are appropriately conducted and assist others learning these methods, and to follow reporting guidelines where available. While there are currently no EQUATOR (Enhancing the QUAlity and Transparency Of health Research) Network reporting guidelines specific to time series analyses, Jandoc et al. [33] have published methodological and reporting recommendations for studies using ITS analysis which provide a good basis.

## Conclusion

ITS analysis, especially when combined with a control series, is a powerful study design for assessing population-level health intervention impacts, and its use is increasing. Segmented regression, the most common method for ITS analysis, is not always adequate. Thus, for researchers interested in ITS analysis, ARIMA modelling is a useful tool, as it can account for underlying trends, autocorrelation and seasonality and allows for flexible modelling of different types of impacts.

## Supplementary Information

**Additional file 1.** Data for reproducing results in this manuscript.

**Additional file 2.** R code for reproducing results in this manuscript.

**Additional file 3.** SAS code for reproducing results in this manuscript.

## Data Availability

The dataset supporting the conclusions of this article is included within its additional files.

## Abbreviations

ACF: autocorrelation function
AR: autoregressive
ARIMA model: Automated regressive integrated moving average model
EQUATOR: Enhancing the QUAlity and Transparency Of health Research
ITS: interrupted time series
MA: moving average
PACF: partial autocorrelation function
RCT: randomised controlled trial

## References

[1] Soumerai SB, Starr D, Majumdar SR (2015). How do you know which health care effectiveness research you can trust? A Guide to Study Design for the Perplexed. Prev Chronic Dis.

[2] Shadish WR, Cook TD, Campbell DT. Experimental and quasi-experimental designs for generalized causal inference. Belmont, CA: Wadsworth/Cengage Learning; 2002.

[3] Lopez Bernal J, Cummins S, Gasparrini A (2018). The use of controls in interrupted time series studies of public health interventions. Int J Epidemiol.

[4] Fretheim A, Zhang F, Ross-Degnan D, Oxman AD, Cheyne H, Foy R (2015). A reanalysis of cluster randomized trials showed interrupted time-series studies were valuable in health system evaluation. J Clin Epidemiol.

[5] Bernal JL, Soumerai S, Gasparrini A (2018). A methodological framework for model selection in interrupted time series studies. J Clin Epidemiol.

[6] Lopez Bernal J, Cummins S, Gasparrini A (2016). Interrupted time series regression for the evaluation of public health interventions: a tutorial. Int J Epidemiol.

[7] Wagner AK, Soumerai SB, Zhang F, Ross-Degnan D (2002). Segmented regression analysis of interrupted time series studies in medication use research. J Clin Pharm Ther.

[8] Lagarde M (2012). How to do (or not to do) … assessing the impact of a policy change with routine longitudinal data. Health Policy Plan.

[9] Beard E, Marsden J, Brown J, Tombor I, Stapleton J, Michie S (2019). Understanding and using time series analyses in addiction research. Addiction..

[10] Hyndman R, Athanasopoulos G. Forecasting: principles and practice. 2nd edition. 2018. https://otexts.com/fpp2/. .

[11] Sun L, Klein EY, Laxminarayan R (2012). Seasonality and temporal correlation between community antibiotic use and resistance in the United States. Clin Infect Dis.

[12] Schaffer A, Muscatello D, Cretikos M, Gilmour R, Tobin S, Ward J (2012). The impact of influenza a(H1N1)pdm09 compared with seasonal influenza on intensive care admissions in New South Wales, Australia, 2007 to 2010: a time series analysis. BMC Public Health.

[13] Mellish L, Karanges EA, Litchfield MJ, Schaffer AL, Blanch B, Daniels BJ (2015). The Australian pharmaceutical benefits scheme data collection: a practical guide for researchers. BMC Res Notes.

[14] Bødkergaard K, Selmer RM, Hallas J, Kjerpeseth LJ, Pottegård A, Skovlund E (2020). Using the waiting time distribution with random index dates to estimate prescription durations in the presence of seasonal stockpiling. Pharmacoepidemiol Drug Saf.

[15] Liboschik T, Fokianos K, Fried R tscount: An R Package for Analysis of Count Time Series Following Generalized Linear Models J Stat Softw 2017;82:1–51.

[16] Dunsmuir WTM, Scott DJ (2015). The glarma package for observation-driven time series regression of counts. J Stat Softw.

[17] Schaffer AL, Buckley NA, Dobbins TA, Banks E, Pearson S-A (2015). The crux of the matter: did the ABC’s catalyst program change statin use in Australia?. Med J Aust.

[18] Schaffer AL, Buckley NA, Cairns R, Pearson S-A (2016). Interrupted time series analysis of the effect of rescheduling alprazolam in Australia: taking control of prescription drug use. JAMA Intern Med.

[19] Young JM, Stacey I, Dobbins TA, Dunlop S, Dessaix AL, Currow DC (2014). Association between tobacco plain packaging and Quitline calls: a population-based, interrupted time-series analysis. Med J Aust.

[20] Gilmour S, Degenhardt L, Hall W, Day C (2006). Using intervention time series analyses to assess the effects of imperfectly identifiable natural events: a general method and example. BMC Med Res Methodol.

[21] Lane TJ, Gray S, Hassani-Mahmooei B, Collie A (2018). Effectiveness of employer financial incentives in reducing time to report worker injury: an interrupted time series study of two Australian workers’ compensation jurisdictions. BMC Public Health.

[22] Sun P, Chang J, Zhang J, Khaler K (2011). Evolutionary cost analysis of valsartan initiation among patients with hypertension: a time series approach. J Med Econ.

[23] Box GEP, Jenkins GM, Reinsel GC (2008). Time series analysis: forecasting and control.

[24] Helfenstein U (1991). The use of transfer function models, intervention analysis and related time series methods in epidemiology. Int J Epidemiol.

[25] Pankratz A (1991). Forecasting with dynamic regression models.

[26] Hyndman R, Kostenko AV. Minimum Sample Size requirements for Seasonal Forecasting Models. Foresight Int J Appl Forecast. 2007;:12–5.

[27] Brett J, Schaffer A, Dobbins T, Buckley NA, Pearson SA (2018). The impact of permissive and restrictive pharmaceutical policies on quetiapine dispensing: evaluating a policy pendulum using interrupted time series analysis. Pharmacoepidemiol Drug Saf.

[28] Hyndman RJ, Khandakar Y (2008). Automatic time series forecasting: the forecast package for R. J Stat Softw.

[29] Lu CY, Simon G, Soumerai SB Counter-Point: Staying Honest When Policy Changes Backfire Med Care 2018;56:384. 10.1097/MLR.0000000000000897 PMC5898649 29634631

[30] Shaw J, Murphy AL, Turner JP, Gardner DM, Silvius JL, Bouck Z (2019). Policies for Deprescribing: an international scan of intended and unintended outcomes of limiting sedative-hypnotic use in community-dwelling older adults. Healthc Policy Polit Sante.

[31] Briesacher BA, Soumerai SB, Zhang F, Toh S, Andrade SE, Wagner JL (2013). A critical review of methods to evaluate the impact of FDA regulatory actions. Pharmacoepidemiol Drug Saf.

[32] Hudson J, Fielding S, Ramsay CR (2019). Methodology and reporting characteristics of studies using interrupted time series design in healthcare. BMC Med Res Methodol.

[33] Jandoc R, Burden AM, Mamdani M, Lévesque LE, Cadarette SM (2015). Interrupted time series analysis in drug utilization research is increasing: systematic review and recommendations. J Clin Epidemiol.

